# Is carriage of multidrug-resistant organisms a risk factor for nosocomial infections in an infectious diseases ICU?

**DOI:** 10.1186/cc14173

**Published:** 2015-03-16

**Authors:** M Lupse, M Flonta, L Herbel, A Petrovan, A Binder, N Todor, A Cioara

**Affiliations:** 1 University of Medicine and Pharmacy, Cluj-Napoca, Romania; 2 Teaching Hospital of Infectious Diseases, Cluj-Napoca, Romania; 3 'Ion Chiricuta' Institute of Oncology, Cluj-Napoca, Romania

## Introduction

The objective was to evaluate whether asymptomatic carriage of methicillin-resistant *Staphylococcus aureus *(MRSA), vancomycin-resistant enterococci (VRE) and extended-spectrum betalactamase producing Gram-negative bacilli (ESBL-GN) on admittance to the ICU of the University Hospital of Infectious Diseases Cluj-Napoca, Romania is a risk factor for infection due to these multidrug-resistant organisms (MDRO) during hospitalization.

## Methods

A prospective study during a 6-month period (June to November 2014), including all adult patients admitted to our ICU. All patients were screened on admittance for nasal MRSA, intestinal VRE and ESBL-GN carriage. Patients admitted with any localization of infections due to these organisms were excluded. Patients were monitored for developing nosocomial infections due to MDRO during hospitalization. We evaluated previous colonization as a risk factor for future infections. We used bioMerieux selective chromogenic media for MDRO for screening and Vitek2Compact for identification. Statistical analysis was performed with chi-square test and univariate analysis.

## Results

From 119 screened adult patients, 65 women (54.6%), average age 67 years, we had at screening on admittance into the ICU: 14 positive MRSA (11.8%), 63 positive ESBL-GN (52.9% - 41 strains of *Escherichia coli*, 26 strains of *Klebsiella *sp., 11 strains of *Proteus mirabilis *and one strain of *Enterobacter cloacae*) and 35 positive VRE (29.4% - 33 strains of *Enterococcus faecium *and two strains of *Enterococcus faecalis*) without concomitant infection with these MDRO. The average duration of ICU stay was 7.32 days. During hospitalization, 14 patients (11.8%) developed nosocomial infections due to MDRO. Colonization with MDRO has preceded nosocomial infections in: one of four patients with MRSA-positive blood cultures (*P *= 0.96), seven of eight patients with ESBL-GN infections (*P *= 0.10) and three of four patients with VRE urinary tract infections (*P *= 0.14). Although not statistically significant, owing to the low number, most patients who developed infections with ESBL-GN had previous intestinal colonization.

## Conclusion

The carriage of MDRO in ICU-admitted patients is important, especially for ESBL-GN. The incidence of nosocomial infections with MDRO in the ICU is high. ESBL-GN intestinal colonization could be a risk factor for nosocomial infections but further studies are needed to confirm this.

